# NETRIN-4 Protects Glioblastoma Cells FROM Temozolomide Induced Senescence 

**DOI:** 10.1371/journal.pone.0080363

**Published:** 2013-11-12

**Authors:** Li Li, Yizhou Hu, Irene Ylivinkka, Huini Li, Ping Chen, Jorma Keski-Oja, Marko Hyytiäinen

**Affiliations:** 1 Departments of Virology and Pathology, Faculty of Medicine, the Haartman Institute, Translational Cancer Biology Research Program and Helsinki University Hospital, University of Helsinki, Helsinki, Finland; 2 Department of Oncology, the Second Clinical College, Harbin Medical University, Harbin, People's Republic of China; 3 Research Programs Unit, Genome-Scale Biology and Institute of Biomedicine, University of Helsinki, Helsinki, Finland; University of Pennsylvania School of Medicine, United States of America

## Abstract

Glioblastoma multiforme is the most common primary tumor of the central nervous system. The drug temozolomide (TMZ) prolongs lifespan in many glioblastoma patients. The sensitivity of glioblastoma cells to TMZ is interfered by many factors, such as the expression of O-6-methylguanine-DNA methyltransferase (MGMT) and activation of AKT signaling. We have recently identified the interaction between netrin-4 (NTN4) and integrin beta-4 (ITGB4), which promotes glioblastoma cell proliferation via activating AKT-mTOR signaling pathway. In the current work we have explored the effect of NTN4/ITGB4 interaction on TMZ induced glioblastoma cell senescence. We report here that the suppression of either ITGB4 or NTN4 in glioblastoma cell lines significantly enhances cellular senescence. The sensitivity of GBM cells to TMZ was primarily determined by the expression of MGMT. To omit the effect of MGMT, we concentrated on the cell lines devoid of expression of MGMT. NTN4 partially inhibited TMZ induced cell senescence and rescued AKT from dephosphorylation in U251MG cells, a cell line bearing decent levels of ITGB4. However, addition of exogenous NTN4 displayed no significant effect on TMZ induced senescence rescue or AKT activation in U87MG cells, which expressed ITGB4 at low levels. Furthermore, overexpression of ITGB4 combined with exogenous NTN4 significantly attenuated U87MG cell senescence induced by TMZ. These data suggest that NTN4 protects glioblastoma cells from TMZ induced senescence, probably via rescuing TMZ triggered ITGB4 dependent AKT dephosphorylation. This suggests that interfering the interaction between NTN4 and ITGB4 or concomitant use of the inhibitors of the AKT pathway may improve the therapeutic efficiency of TMZ.

## Introduction

Netrin-4 is a secreted laminin-related protein, which was originally observed to guide axons  during neuronal development [1–3]. Recently, it has been found to be expressed in many other tissues and tumor types, and to contribute to the regulation of cell adhesion, migration, proliferation, and apoptosis [4–8]. In the central nervous system, NTN4 is strongly expressed by astrocytes [1,2]. In glioblastoma, high concentrations of NTN4 decrease cell proliferation in cultured glioblastoma cells. Interestingly, the expression of NTN4 is down-regulated when compared to normal brain tissue. However, low concentrations of NTN4 promote glioblastoma cell proliferation via integrin beta-4 signaling. Furthermore, NTN4 is expressed at higher levels in the white matter-invading glioblastoma cells than in the tumor cores [9].

Glioblastoma multiforme is the most common primary tumor of the central nervous system [10,11]. Its median survival period is less than 15 months after the diagnosis [12]. Although there are no curative treatments for this fatal disease, the therapeutic efficacy for temozolomide (TMZ), an orally taken alkylating agent, has been verified in the treatment of glioblastoma [13,14]. By combining radiotherapy with TMZ, patients had significantly longer survival time after diagnosis [15,16]. 

The therapeutic function of temozolomide is based on its capacity to methylate DNA [17,18], which most often causes cellular cytotoxicity by forming O6-methylguanine adducts. During DNA replication, O6-methylguanine mispairs with thymine [19]. This mismatch subsequently activates endless futile cycles of the mismatch repair (MMR) system due to the irreplaceable methylated adduct, leading to single- and double-strand breaks in DNA. Eventually, these DNA strand breaks trigger cellular senescence and mitotic arrest in tumors [18,20].

The therapeutic benefit of temozolomide on glioblastoma is interfered by at least two factors. First, the sensitivity of glioblastoma cells to TMZ is inhibited by the expression of O-6-methylguanine-DNA methyltransferase (MGMT) [21,22]. MGMT is a DNA-repair enzyme, which can remove methylated DNA adducts, thereby abolishing TMZ induced DNA damage and cell death. Glioblastoma patients with high expression of MGMT have usually minimal response to temozolomide [23]. Second, the therapeutic efficacy of temozolomide depends on the activation of AKT, a major regulator of tumorigenesis. Substantial activation of AKT occurs in a high percentage of glioblastomas, which is primarily due to the deletion or inactivation of PTEN [24]. AKT phosphorylation suppresses temozolomide-induced glioma cell senescence via its various downstream survival signals [25–27]. Combined treatment of the AKT inhibitor and temozolomide has additive effects on glioma and melanoma treatment [28,29].

 Among a number of molecular interactions, which construct an extensive and complicated network to modulate the activation of AKT [30–32], we found recently an interaction between NTN4 and ITGB4, which stimulates AKT phosphorylation [33]. Interestingly, the silencing of ITGB4 can induce cellular senescence in various cells types [34–36]. Therefore, the NTN4/ITGB4 transduced AKT activation possibly influences TMZ triggered glioblastoma cell senescence. We describe here the effects of NTN4-ITGB4 interaction on TMZ induced glioblastoma cell senescence and clarify the underlying molecular mechanisms.

## Materials and Methods

Immunoblotting analysis, transfection of cells, total RNA extraction, bioinformatics analysis, reverse transcription, and Real-time Reverse Transcription–PCR were performed as described [33,37].

### Cell Lines and Reagents

U251MG (Health Science Research Resources Bank, Osaka, Japan), U118MG, T98G, U87MG (American Type Culture Collection, Rockville, MD) and 293FT cells (Invitrogen Life Technology, Carlsbad, CA) were cultured according to the supplier’s instructions. 

The following primary antibodies and recombinant protein were used: anti-ITGB4 from Sigma-Aldrich (St Louis, MO); anti- AKT, anti–p44/43MAPK (ERK1/2), anti-mammalian target of rapamycin (mTOR), anti-phosphor AKT (Ser473), anti-phospho-p44/43MAPK (ERK1/2) (Thr202/Tyr204), and anti-phospho-mammalian target of rapamycin (mTOR; Ser2448) from Cell Signaling (Danvers,MA); and anti–β-tubulin from Santa Cruz Biotechnology (Santa Cruz, CA) and recombinant NTN4 from R&D Systems (Minneapolis, MN).

### TMZ treatment

Temozolomide (Sigma Chemical Co., St. Louis, MO) was dissolved in DMSO. The cells were treated with TMZ [100 μM] for 3 h, with the final concentration of DMSO not exceeding 0.1% (v/v). Subsequently, the cells were gently washed twice with PBS, and then cultured in Dulbecco modified Eagle medium (DMEM) containing 10% fetal calf serum (FCS) for the indicated periods of time for further experiments.

### Lentiviral Silencing/Overexpression of Gene Expression

Plasmids expressing shRNAs targeted against the indicated gene were obtained from The RNAi Consortium through Biomedicum Helsinki Functional Genomics Unit (FuGU) (hairpin-pLKO.1 vector). Five different constructs for each gene were tested. A nontargeting construct was used as control for nonspecific effects (NT-pLKO.1 vector). Plasmids harboring the indicated gene were constructed as described [33,37]. pLVX-puro empty vector was used as a control. To produce recombinant lentivirus particles, 293FT producer cells were cotransfected with the packaging/envelope plasmids (pCMVdr8.74 and pMD2-VSVG; Addgene, Cambridge, MA) and target plasmids by using the Lipofectamine (Invitrogen) transfection method. Normal complete culture medium (high-glucose Dulbecco modified Eagle medium, DMEM) was changed to the 293FT cells 24 h after transfection. Subsequently, 48 h later, the viral supernatants were harvested and filtered through a 0.45-μm filter. The titer of the virus was investigated. After a 24-hour infection with virus, the supernatants were replaced with complete medium (DMEM) for the subsequent assays. For stable shRNA expression, the infected U251MG cells were subjected to selection with 2 μg/ml puromycin for 72 h. The efficiency of the transduction was measured by monitoring the indicated gene expression with Q-RT-PCR.

### Senescence-Associated Beta-Galactosidase Staining (SA β-gal Staining)

Cells were cultured in 24-well plate at densities of 3000~ 5000 cells per well for the indicated times. Subsequently, the cells were washed once with phosphate buffered saline (PBS, 137 mM NaCl, 2.7 mM KCl, 10 mM Na_2_HPO_4_, 2 mM KH_2_PO_4_), and then fixed in PBS containing 0.5% glutaraldehyde at room temperature for 20 min. After two PBS washes, the cells were incubated with SA β-gal substrate solution (1 mg/ml X-gal, 40 mM citric acid-sodium phosphate buffer, pH 6.0, containing 5 mM potassium ferricyanide, 5 mM potassium ferrocyanide, 150 mM NaCl, and 2 mM MgCl_2_) at 37°C in dark for 16-20 h [38]. The reaction was terminated by removing SA β-gal substrate solution and washing twice with PBS. The cells were stored in 70% glycerol at 4 °C. The cells were finally photographed by using Axiovert 200 inverted epifluorescence microscope (Carl Zeiss). The ratio of blue color stained cells was analyzed with the ImageJ program (National Institutes of Health, Bethesda, MD).

### Statistical Analysis

All numerical values represent mean ± SE or SD. Statistical significance was determined with the nonparametric Mann-Whitney U test. 

## Results

### Silencing of either ITGB4 or NTN4 induces glioblastoma cell senescence

**Figure 1:**
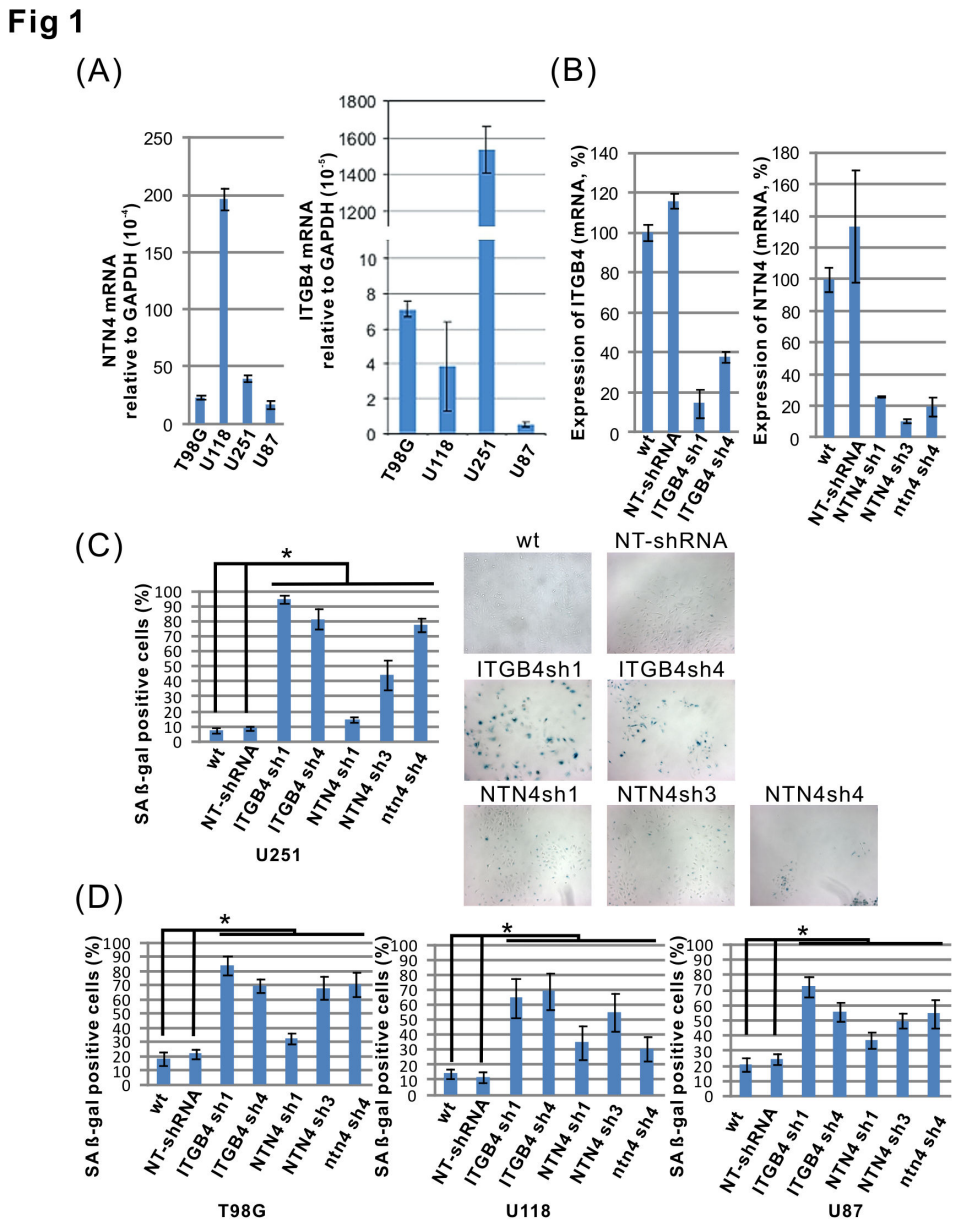
Silencing of the expression of either NTN4 or ITGB4 induces glioblastoma cell senescence. The expression levels of NTN4 and ITGB4 were determined in four glioblastoma cell lines by using Q-RT-PCR (A). U251MG cells were infected by lentivirus harboring shRNAs of target genes and nontargeting control shRNA (NT-shRNA). The silencing efficiencies of each shRNA were confirmed by Q-RT-PCR (B). Seven days after infection, U251MG cells were fixed and analyzed by the senescence assay. Silencing of either NTN4 or ITGB4 increased the number of cells undergoing senescence (blue color) in beta-gal staining (C). Similar results were observed in T98G, U118, and U87MG cells (D). Mean ± SE, n ≥ 3, * p-value < 0.05.

We have previously analyzed the expression of NTN4 and ITGB4 genes in the U251MG and U87MG glioblastoma cell lines. By Q-RT-PCR, we confirmed that both NTN4 and ITGB4 were expressed in all four analyzed glioblastoma cell lines, namely U251MG, U87MG, U118MG and T98G. Interestingly, the expression of ITGB4 was much lower in U87MG than in the other three cell lines (Figure 1A). To understand the effects of NTN4 and ITGB4 on glioblastoma cell senescence, we inhibited the expression of these two genes in U251MG cells by using short hairpin RNAs (shRNAs). The expression levels of NTN4 and ITGB4 genes were measured by Q-RT-PCR. The results indicated that three NTN4 and two ITGB4 shRNAs efficiently reduced the expression in U251MG cells (Figure 1B). We then detected the number of senescent cells by using senescence-associated beta-galactosidase assay. We observed that silencing of either NTN4 or ITGB4 significantly induced U251MG cell senescence (Figure 1C). Similar results were obtained by silencing the expression of either NTN4 or ITGB4 in U118MG, T98G and U87MG cells (Figure 1D).

### TMZ induces cell senescence in U251MG and U87MG cells

**Figure 2:**
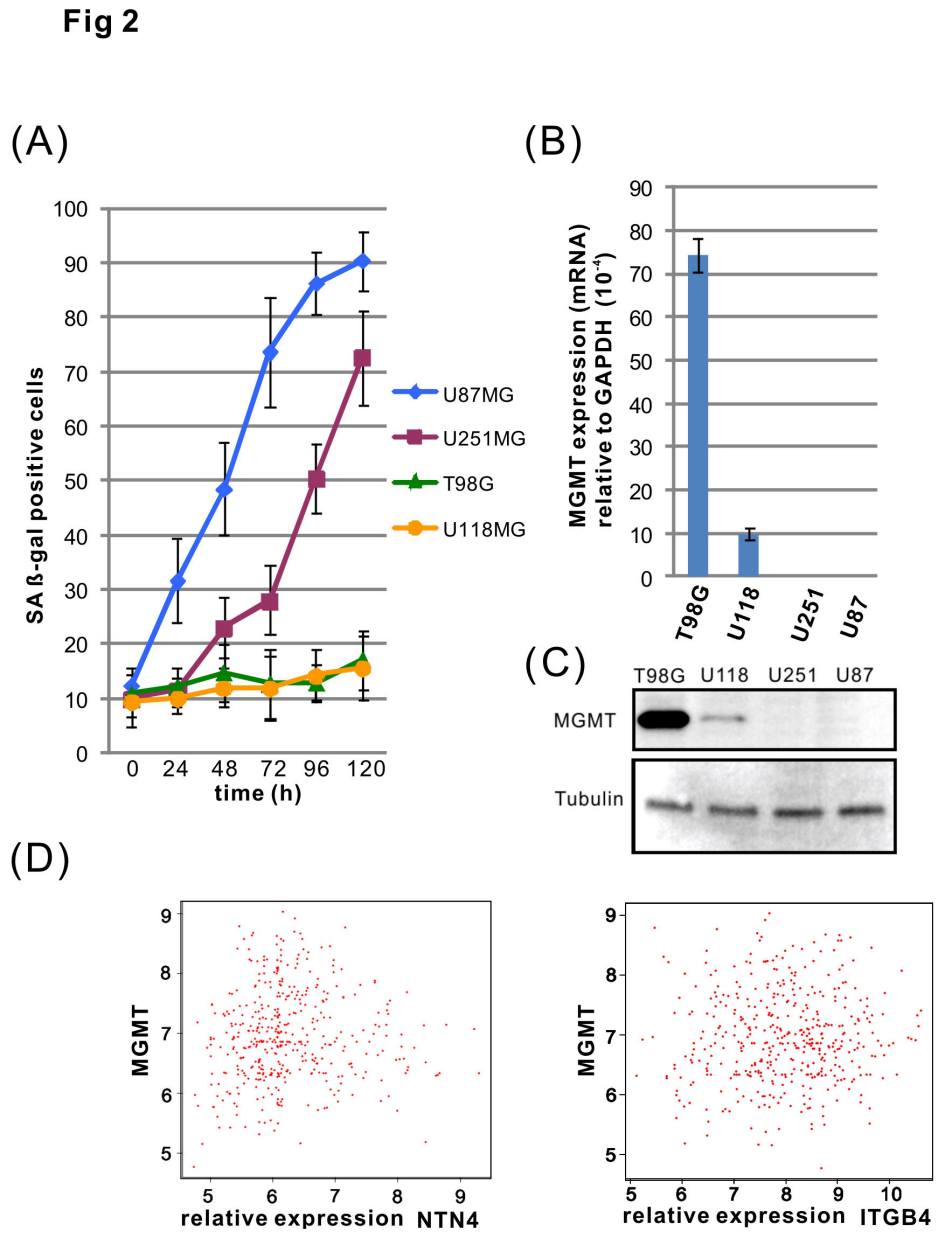
U87MG and U251MG glioblastoma cells are sensitive to TMZ induced senescence. T98G, U118MG, U251MG and U87MG cells were treated with TMZ [100μM] for 3 h, and then cultured in medium containing 10% FCS. The cells were subsequently subjected to a time-course cell senescence assay. The cells were fixed every 24 h and the cell senescence rate was analyzed by beta-gal assay. U87MG cells were observed to be the most sensitive cell line to TMZ, as the percentage of senescence increased already 24 h after TMZ treatment. The U251MG cells responded more slowly, and started to undergo senescence about 48 h after the treatment. In contrast, the T98G and U118MG cells were insensitive to TMZ induced senescence (A). The expression of MGMT in the four glioblastoma cell lines was determined by using both Q-RT-PCR and Western blotting. MGMT was highly expressed in T98G and U118 cells, but undetectable in U251MG and U87MG cells (B, C).  On the basis of 425 primary GBM tumors from TCGA, we calculated the Pearson product-moment correlation coefficient. Bioinformatics analysis revealed that the expression of MGMT does not correlate with the expression of either NTN4 (r value=0.041) or ITGB4 (r value=0.043) (D).  Mean ± SE, *n* ≥ 3.

We assessed the TMZ sensitivity of four glioblastoma cell lines by using a cell senescence assay. U87MG cells started to undergo senescence about 24 h after treatment with TMZ, and more than 70% of the cells underwent senescence after 72 h. U251MG cells displayed signs of senescence after 48 h and more than 70% cells were senescent by 5 days. However, T98G and U118MG cells showed little or only negligible sensitivity to TMZ (Figure 2A). We observed next the expression of MGMT in the cell lines using both Q-RT-PCR and immunoblotting. We found that MGMT is highly expressed in the T98G and U118MG cells, but not in U251MG or U87MG cells (Figure 2B, C). This result is consistent with the previous reports from other groups [39–42], and suggests that MGMT provides resistance to TMZ in T98G and U118MG cells. To investigate whether the expression levels of MGMT and NTN4/ITGB4 are associated in glioblastoma tissues, we analyzed the expression levels of primary tumors from The Cancer Genome Atlas - Glioblastoma multiforme (TCGA-GBM) repository. The expression of MGMT does not correlate with the expression of either NTN4 or ITGB4 (Figure 2D). This suggests that the biological function of either NTN4 or ITGB4 is independent of the expression of MGMT. Although two MGMT negative cell lines (U251MG and U87MG) responded to TMZ treatment, U87MG cells were more sensitive towards TMZ than U251MG cells. Therefore, we used U87MG and U251MG cells to investigate the effects of NTN4/ITGB4 interaction on TMZ induced cell senescence.

### NTN4 rescues TMZ induced senescence and AKT dephosphorylation in U251MG, but not in U87MG cells

**Figure 3:**
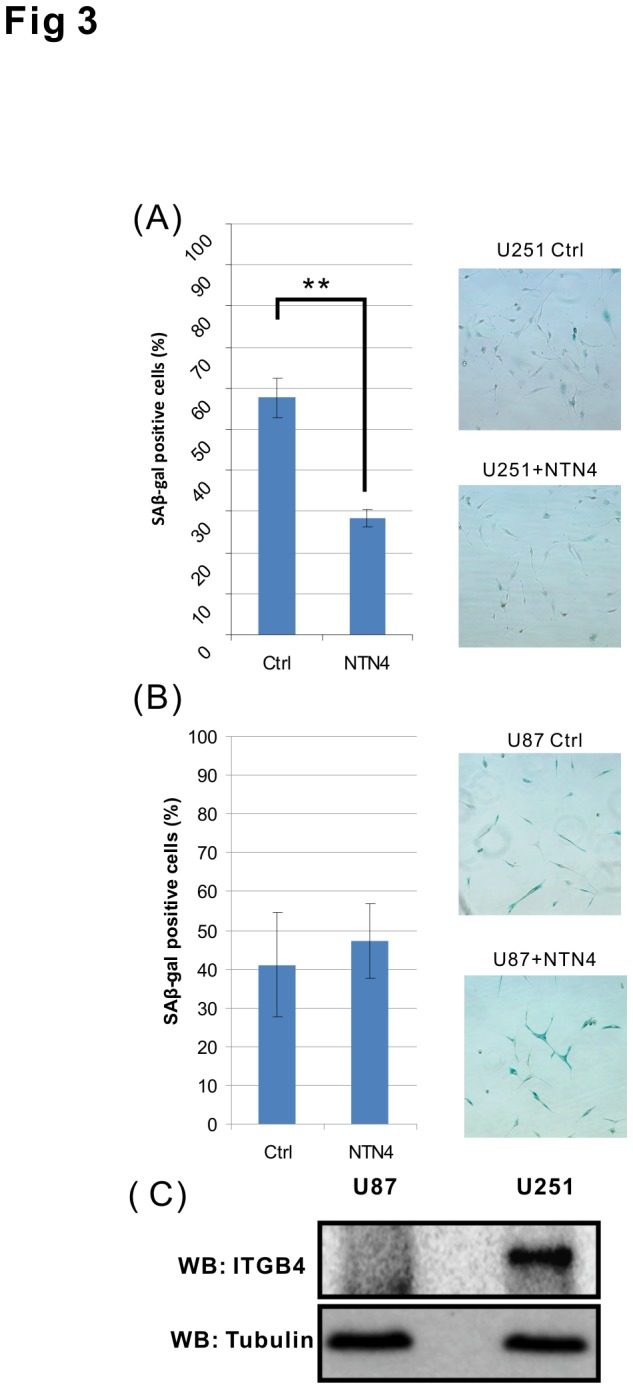
NTN4 partially prevents TMZ induced cell senescence of U251MG, but not of U87MG cells. U251MG and U87MG cells were treated with TMZ [100μM] for 3 h, and then cultured in medium containing 10% FCS and recombinant NTN4 protein [100 ng/ml] as indicated. After 4 d (U251MG) or 2 d (U87MG) the cells were subjected to beta-gal staining. NTN4 reduced the senescence rate of TMZ treated U251MG cells (A), but had no significant effects on U87MG cells (B). Both U87MG and U251MG cells were cultured to about 50% confluence, and then lysed for western blotting (WB) analysis. ITGB4 protein was observed in U251MG cells, but was barely detectable in U87MG cells. Tubulin was used as a loading control (C). Mean ± SE, *n* ≥ 3, ** p-value < 0.01.

**Figure 4:**
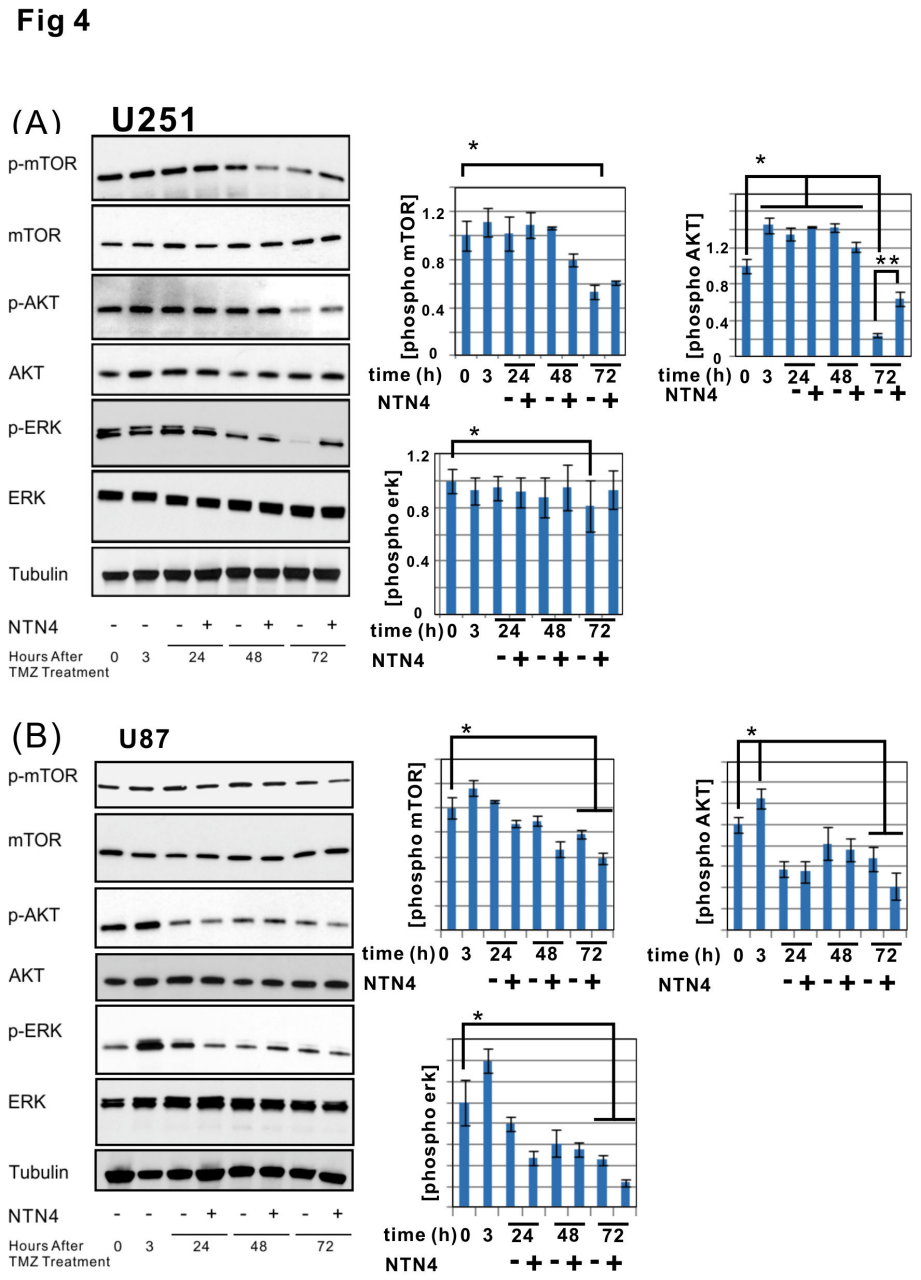
NTN4 partially rescues AKT and ERK phosphorylation in TMZ treated U251MG cells, but not in TMZ treated U87MG cells. U251MG and U87MG cells were treated with TMZ [100μM] for 3 h, and then cultured in medium containing 10% FCS supplemented with recombinant NTN4 protein [100 ng/ml]. Recombinant NTN4 protein was resupplied every 24 h. The cells were lysed for immunoblotting before (0 h) or after TMZ treatment (3 h), and every 24 h after TMZ treatment (24 h, 48 h, 72 h), total mTOR, total AKT, total ERK, and tubulin were used as loading control. In both U251MG and U87MG cell lines, the levels of p-AKT increased immediately after TMZ treatment and were significantly reduced 72 h later, while p-ERK (marked by arrowhead) was continuously inhibited after TMZ treatment. 72 h after TMZ treatment, NTN4 partially rescued AKT and ERK phosphorylation in U251MG cells (A), but not in U87MG cells (B).

Next we explored the effects of exogenous recombinant NTN4 on TMZ induced senescence. NTN4 significantly inhibited U251MG cell senescence induced by TMZ (Figure 3A). However, the addition of exogenous NTN4 did not display any obvious influence on the senescence rate of TMZ -treated U87MG cells (Figure 3B). U87MG cells expressed ITGB4 at a much lower level than U251MG cells, both at mRNA (Figure 1A) and protein levels (Figure 3C). Thus, the low expression of ITGB4 in U87MG cells may impair signal transduction from NTN4 to AKT. We then analyzed the time scale of the stimulatory effects of NTN4 on AKT and ERK signaling pathways in both U87MG and U251MG cell lines after TMZ treatment. Unexpectedly, the phosphorylation of AKT was induced immediately after TMZ treatment both in U87MG and U251MG cells. This phosphorylation effect of AKT lasted for 48 h, and was then severely reduced 72 h later. In contrast, the phosphorylation of ERK was constantly attenuated in both U251MG and U87MG cells after the treatment. Accordingly, 72 h after TMZ treatment, the phosphorylation of AKT, ERK and mTOR, an AKT downstream effector, were significantly rescued by NTN4 in U251MG, but not in U87MG cells (Figures 4A and 4B). These results indicated that the NTN4-AKT signaling transduction was at least partially disturbed in U87MG cells.

### NTN4 prevents TMZ induced cellular senescence in U87MG cell overexpressing ITGB4

**Figure 5:**
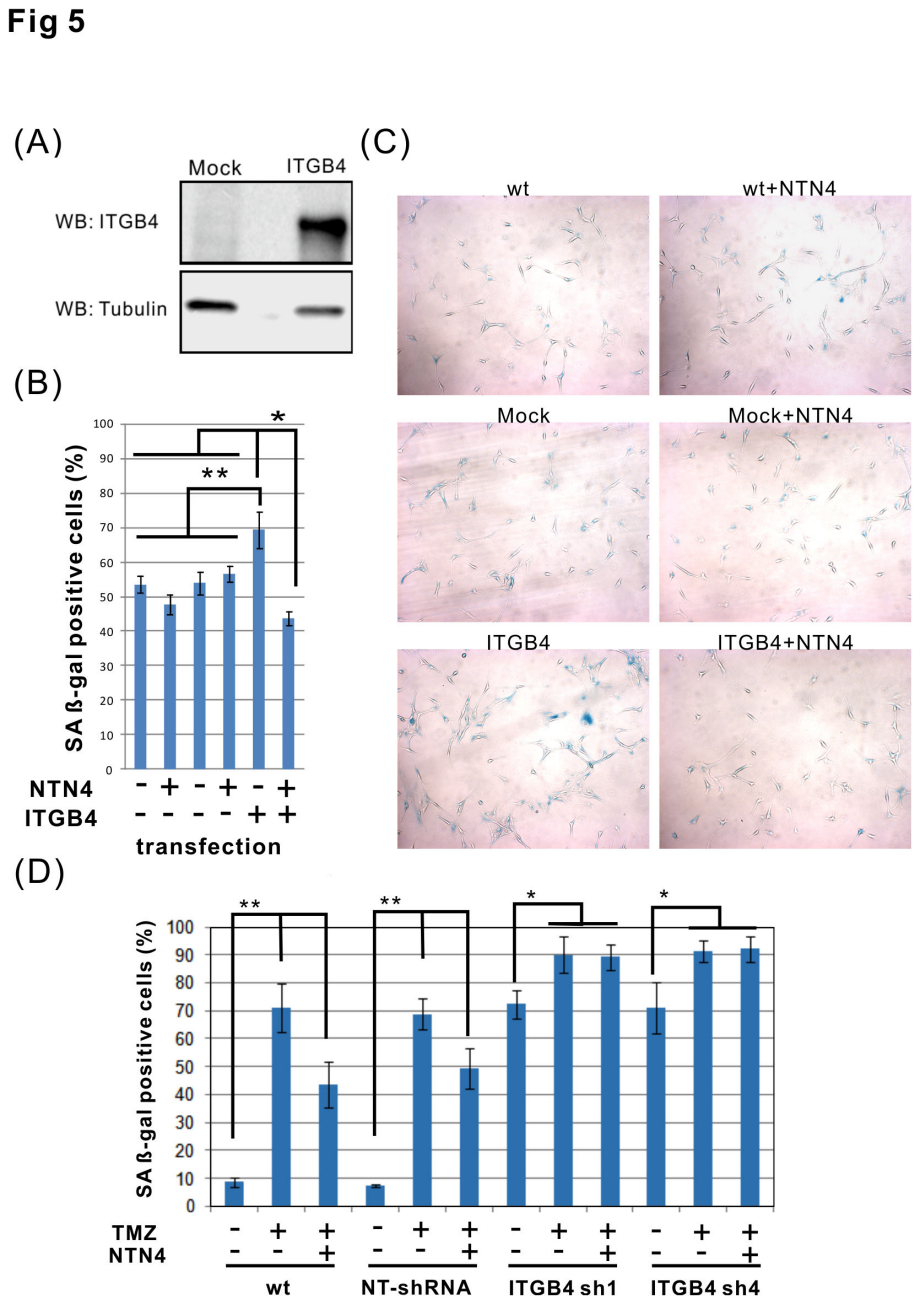
Overexpression of ITGB4 combined with exogenous NTN4 partially prevents TMZ- induced U87MG cell senescence . U87MG cells were transfected to overexpress ITGB4. The expression level of ITGB4 in U87MG was confirmed by immunoblotting (A). ITGB4 overexpressing U87MG cells were treated with TMZ [100 μM] for 3 h, and then cultured in medium containing 10% FCS supplemented by recombinant NTN4 protein [100 ng/ml]. After 2 d, the cells were analyzed for senescence by beta-gal staining. Wild type U87MG and empty vector (Mock) transfected U87MG cells were used as controls. In either wild type U87MG or mock transfected cells, NTN4 displayed no significant effects on TMZ induced cell senescence. In the absence of exogenous addition of recombinant NTN4, overexpression of ITGB4 enhanced TMZ induced U87MG cell senescence, whereas in the presence of recombinant NTN4, the cells overexpressing ITGB4 were partially resistant to TMZ induced cell senescence (B, C). U251MG cells were infected by lentivirus harboring ITGB4 shRNAs and nontargeting control shRNA (NT-shRNA). Three days after infection, U251MG cells were treated with TMZ [100μM] for 3 h, and then cultured in medium containing 10% FCS and recombinant NTN4 protein [100 ng/ml]. Four days later, U251MG cells were fixed and analyzed by beta-gal staining. Silencing of ITGB4 increased the number of U251MG cells undergoing senescence. NTN4 reduced the senescence rate of wild type or non-targeting control U251MG cells induced by TMZ treatment, but NTN4 did not affect ITGB4-silenced U251MG cells (D). Mean ± SE, n ≥ 3, **p value < 0.01, * p value < 0.05.

We assessed next the role of NTN4 in U87MG cells overexpressing ITGB4. We generated U87MG cells overexpressing ITGB4, and confirmed the expression by immunoblotting (Figure 5A). These cells were then used for senescence-associated beta-galactosidase assay. Neither in wild type U87MG cells nor in mock transfected cells did the addition of exogenous recombinant NTN4 display any significant effect on TMZ induced cellular senescence. Interestingly, overexpression of ITGB4 aggravated the cytotoxicity of TMZ on U87MG cells, but this aggravation was decreased by endogenous NTN4. Furthermore, NTN4 delayed the TMZ induced cellular senescence in U87MG cells overexpressing ITGB4, compared with either wild type or empty vector control cells (Figures 5B and 5C). Furthermore, we investigated the effects of NTN4 and TMZ on ITGB4-silenced U251MG cells. Silencing of ITGB4 increased the number of U251MG cells undergoing senescence. Although NTN4 reduced the senescence rate of wild type or non-targeting control U251MG cells induced by TMZ treatment, NTN4 did not affect ITGB4-silenced U251MG cells (Figure 5D). This result indicates that ITGB4 mediates the protective effect of NTN4 on TMZ treated glioblastoma cells.

## Discussion

NTN4 was originally identified as an axon guidance cue. Besides its role in attracting and/or repelling axons during neural development, NTN4 is highly expressed in astrocytes, and it enhances the mitotic ability of neural stem cells through forming a molecular complex with laminin gamma1 chain, and integrin alpha6beta1 [2]. In glioblastomas NTN4 is expressed by the tumor cells at the invading edge, and it promotes glioblastoma cell proliferation via integrin beta-4 mediated AKT-mTOR signaling [9,33]. 

We find here that suppression of the expression of either ITGB4 or NTN4 in different glioblastoma cell lines leads to cellular senescence, consistently with the results from other ITGB4 silenced cell types [34–36]. This suggests that the interaction between NTN4 and ITGB4 can regulate glioblastoma cell senescence. AKT is an integral part of a pivotal signaling pathway, which promotes tumor cell proliferation and protects cells from apoptosis and senescence via a variety of downstream pathways, such as mammalian target of rapamycin (mTOR), glycogen synthase kinase 3 (GSK-3) and Forkhead box O (FOXO) [32,43]. Accumulating experimental evidence indicates that substantial activation of the PI3K/AKT signaling contributes to tumor cell resistance to conventional chemotherapy [44–47]. In glioblastomas, AKT activation can attenuate the therapeutic efficiency of temozolomide both in vitro and in vivo [25,28,48,49]. Because NTN4/ITGB4 may together activate AKT, we investigated the role of this signaling pathway on the temozolomide resistance of glioblastoma cells. To exclude the interference of MGMT on TMZ resistance, we selected two MGMT negative glioblastoma cell lines (U251MG and U87MG) for the current study. We observed that NTN4 protected TMZ induced cellular senescence and attenuated TMZ triggered AKT dephosphorylation in U251MG cells, which express high levels of ITGB4. 

U87MG cells were more sensitive to TMZ treatment than U251MG cells. This distant outcome in these two cell lines was due to the lower expression of ITGB4 in U87 cells. This is possibly due to the expression of wild type p53 gene in U87MG cell, which enhances TMZ efficiency [50–52].

Unexpectedly, ITGB4 overexpressing U87MG cells were more sensitive to TMZ than the mock transfected cells. However, this effect was rescued by exogenous NTN4. One possible reason is that ITGB4, in the absence of NTN4, augments oncogenic senescence in U87MG cells, whereas NTN4/ITGB4 signaling protects the cells from the senescence, via increased AKT signaling. 

Current results suggest that NTN4/ITGB4 stimulated AKT activation provides glioblastoma cells with the ability of TMZ resistance. This finding may have clinical significance. Since the approval of TMZ for patients with newly diagnosed glioblastoma, the remedy of surgical resection followed by radiotherapy with concomitant/adjuvant TMZ has become the new standard of first-line treatment for glioblastoma. However, the therapeutic efficiency of TMZ still depends on the expression of MGMT and the activation of AKT signaling [22,25,27]. Our recent results reveal that glioblastoma tumors of most patients express low levels of NTN4 and high levels of ITGB4. This may partially endow glioblastoma cells with the potential for TMZ sensitivity. However, for patients with relatively high expression of NTN4 and ITGB4 in glioblastoma tumors, the prevention of NTN4/ITGB4 interaction or concomitant use of the inhibitors of the AKT pathway may improve the therapeutic benefit of temozolomide.
